# Geographic access to pediatric neurosurgeons in the USA: an analysis of sociodemographic factors

**DOI:** 10.1007/s00381-023-06172-z

**Published:** 2023-10-04

**Authors:** Daniel Farivar, Nicholas J. Peterman, Nakul Narendran, Kenneth D. Illingworth, Teryl K. Nuckols, David Bonda, David L. Skaggs

**Affiliations:** 1 Department of Orthopaedic Surgery, Cedars-Sinai Medical Center, Los Angeles, CA USA; 2 Division of General Internal Medicine, Cedars-Sinai Medical Center, Los Angeles, CA USA; 3 Department of Neurological Surgery, University of Washington Medical Center, Seattle, WA USA

**Keywords:** Geographic access, Pediatric neurosurgery, Socioeconomic, Outcomes

## Abstract

**Purpose:**

Geographic access to physicians has been shown to be unevenly distributed in the USA, with those in closer proximity having superior outcomes. The purpose of this study was to describe how geographic access to pediatric neurosurgeons varies across socioeconomic and demographic factors.

**Methods:**

Actively practicing neurosurgeons were identified by matching several registries and membership logs. This data was used to find their primary practice locations and the distance the average person in a county must travel to visit a surgeon. Counties were categorized into “surgeon deserts” and “surgeon clusters,” which were counties where providers were significantly further or closer to its residents, respectively, compared to the national average. These groups were also compared for differences in population characteristics using data obtained from the 2020 American Community Survey.

**Results:**

A total of 439 pediatric neurosurgeons were identified. The average person in a surgeon desert and cluster was found to be 189.2 ± 78.1 miles and 39.7 ± 19.6 miles away from the nearest pediatric neurosurgeon, respectively. Multivariate analyses showed that higher Rural–Urban Continuum (RUC) codes (*p* < 0.001), and higher percentages of American Indian (*p* < 0.001) and Hispanic (*p* < 0.001) residents were independently associated with counties where the average person traveled significantly further to surgeons.

**Conclusion:**

Patients residing in counties with greater RUC codes and higher percentages of American Indian and Hispanic residents on average need to travel significantly greater distances to access pediatric neurosurgeons.

**Supplementary Information:**

The online version contains supplementary material available at 10.1007/s00381-023-06172-z.

## Introduction

Numerous studies have demonstrated that minority residents and those of lower socioeconomic status suffer from poorer access to healthcare [1–3]. These factors have long been a concern for US policy makers with evidence mounting for a need to address and implement focused change [4, 5]. Just as these patient factors influence outcomes, so do physician characteristics. Several studies have shown that physicians’ conscious and subconscious views on the race, ethnicity, and political orientation of their patients may interfere with the care they deliver [6, 7]. Newer literature is also revealing the role that larger, community level factors play. For example, geographic access to physicians has been shown to be unevenly distributed throughout the USA [8–13], with those in closer proximity to providers having superior health outcomes [14–20].

Importantly, this geographic effect exists across specialties. Wang et al. found that patients with breast cancer traveling further to primary care providers were diagnosed at later stages [21]. Another study identified that patients in rural areas with improved transportation access attended significantly more regular check-ups for chronic conditions [22]. Odisho et al. assessed the geographic distribution of urologists throughout the USA and found that 63% of counties lacked even a single urologist and that urologists were less likely to be found in rural counties compared to metropolitan counties (OR 0.03, 95% CI 0.02–0.06) [11]. Similar results were found in orthopaedic surgery, as the distribution of foot and ankle and hand surgeons were reported to also vary substantially by geographic location [12, 13].

Neurosurgery is another highly specialized field that also suffers from geographic disparities. Rahman et al. showed there are only 1.47 neurosurgeons per 100,000 population (interquartile range, 1.02–2.27) and that a greater supply of these surgeons work in areas positively associated with regional levels of college education, median income, and median age and negatively associated with unemployment, poverty, and percent uninsured [8]. Like many surgical specialties, neurosurgeons can acquire further sub-specialization in pediatrics to treat children with more complex pathologies. Dewan et al. illustrated that globally, low- and middle-income countries suffer the greatest burden of pediatric neurosurgical disease while having the smallest number of pediatric neurosurgeons and appropriate facilities [9]. In this study, we aim to describe how geographic access to pediatric neurosurgeons in the USA varies across socioeconomic and demographic factors.

## Methods

### Populations

A current list of neurosurgeons specializing in pediatrics in the USA was obtained using membership logs in May 2022 from the American Association of Neurological Surgeons (AANS), Congress of Neurological Surgeons (CNS) [23]. Results from this search were matched with data from the National Plan and Provider Enumeration System (NPPES) National Provider Identifier (NPI) registry to identify actively practicing members as well as exact practice locations [24]. It was necessary to match membership logs with the NPI registry because the latter only listed physicians’ general specialty taxonomy (e.g., neurosurgery), whereas the former could identify more detailed specialty taxonomy (e.g., pediatric neurosurgeon).

### Geographic access measures

The Python module Nominatim was used to acquire exact latitudes and longitudes of practice locations, which was subsequently matched with zip code and county level geospatial boundaries from the United States Census Bureau [25, 26]. The distance from the centroid of a zip code area to the nearest pediatric neurosurgeon was then calculated. For all zip codes in a given county, an average surgeon distance weighted by a county’s population size was then found, termed “county-to-surgeon distance.” Extrapolating county distances from zip code distances provided more granular data that helped reduce bias and making inappropriate generalizations about counties.

### Socioeconomic and demographic measures

The socioeconomic and demographic data from the 2020 American Community Survey (ACS) was obtained for each county [27]. The Rural–Urban Continuum (RUC), a 1–9 ranking (with 9 being most rural) designed by the US Department of Agriculture, was obtained for each county and geographic region [28]. Other county variables assessed from the ACS included average age, percentage of population under 18, self-reported race, Area Deprivation Index (ADI), household income, those without health insurance, children with a disability, those with mobile homes, those renting homes, those lacking motor vehicles, those lacking telephones, households with children, those not speaking English, those speaking Spanish, households with computers available, households with Internet, families in poverty, median rent, unemployment rate, average family size, education, and veteran population size. Political leanings of each county were also estimated using the 2020 presidential precinct data for the 2020 general election reported by the New York Times [29], calculated as the percentage of Republican voters from only those who voted Republican or Democrat, excluding any third political parties in the USA.

### Analyses

Moran’s *I* cluster analyses were performed to determine statistically significant, *p* < 0.05, spatial variation on the average distance to the nearest pediatric neurosurgeon for each county. When distances to the nearest neurosurgeon were significantly greater than the national average for both a county of interest and the average of all its neighboring counties, this county was termed as “surgeon desert.” In contrast, when these distances were less than the national average for both a county and the average of its neighbors, the county was termed as “surgeon cluster.” A neighbor is defined as any adjacent county whose centroid is within a set distance to the county of interest’s own centroid. The set distance is defined as the minimum distance for all counties to have at least one neighbor, which is about 90 miles. Recorded socioeconomic and geographic access variables were then compared between the surgeon deserts and surgeon clusters using a two-tailed *T*-test. Adjusted analyses were performed using a geospatially weighted multivariable linear regression to evaluate the independent effects of demographic and socioeconomic county characteristics on travel distance to providers. Regression covariates were iteratively chosen using an algorithm to optimize the predictive value of the regression and minimize covariance. As such, covariates with *p* > 0.1 were eliminated from the regression. No tests to determine study size were performed as this was primarily a descriptive study. If certain geographic areas were missing data they were excluded from analyses.

## Results

**Fig. 1:**
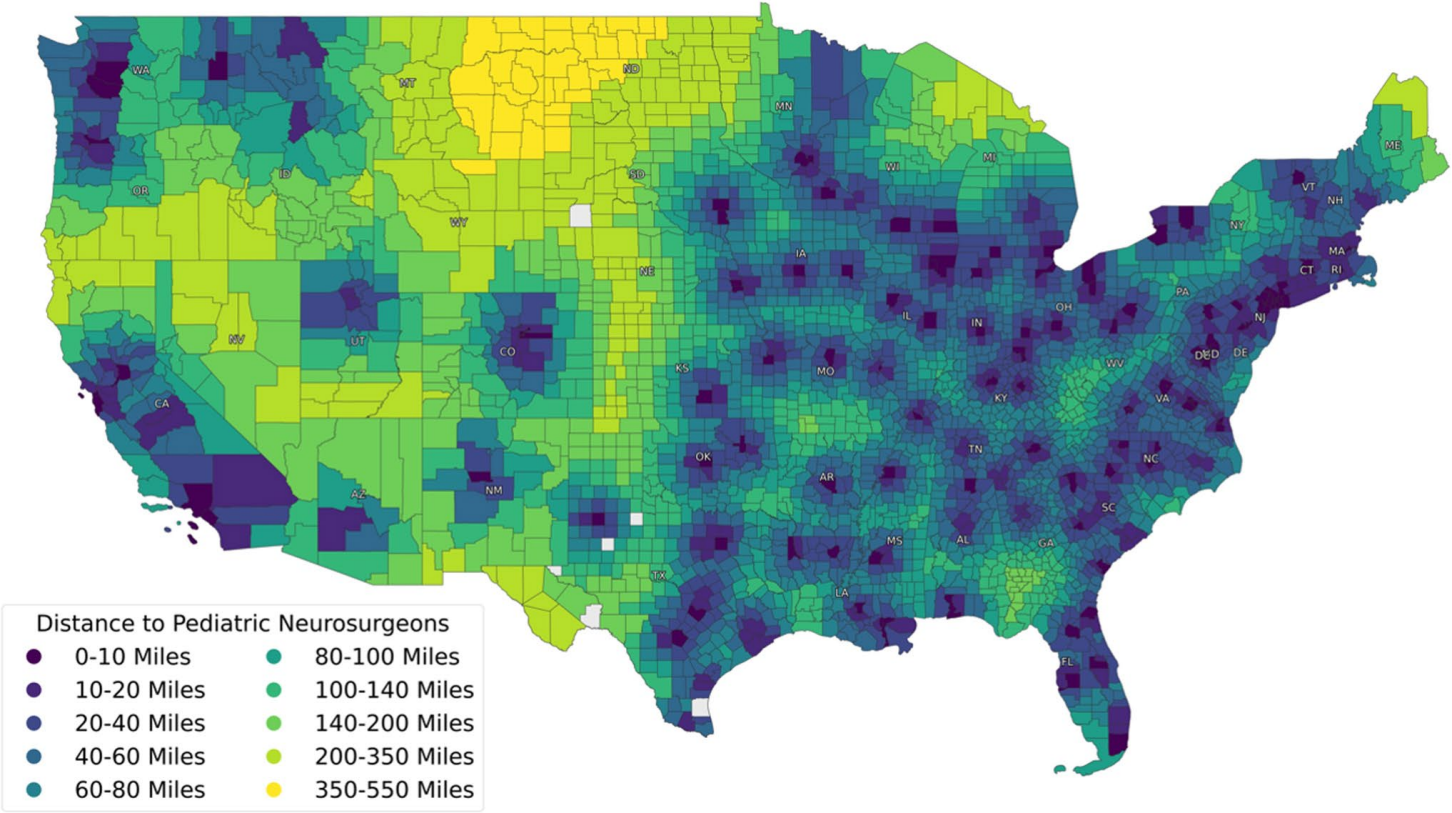
The average distance in miles from any point in a county to the nearest pediatric neurosurgeon displayed on a by-county level. All counties in continental USA were included in the analysis except for a single county in South Dakota (due to missing data)

A total of 439 pediatric neurosurgeons were identified from membership logs. Twenty-three (46.9%) states were found to have 5 or fewer pediatric neurosurgeons, including North Dakota, Wyoming, and Nevada which were found to have none (Table 1). The average “county-to-surgeon” distances are presented in Fig. 1.

**Table 1:** Total number of pediatric neurosurgeons for the 48 contiguous states in the USA and the District of Columbia

**State**	**Total pediatric neurosurgeons**
**North Dakota**	0
**Wyoming**	0
**Nevada**	0
**Idaho**	0
**Vermont**	1
**Rhode Island**	1
**West Virginia**	1
**Maine**	2
**South Dakota**	2
**Delaware**	2
**Montana**	2
**Nebraska**	3
**Connecticut**	3
**New Mexico**	3
**New Hampshire**	3
**Mississippi**	4
**Kansas**	4
**Arkansas**	4
**Kentucky**	4
**Iowa**	5
**South Carolina**	5
**Arizona**	5
**Louisiana**	5
**District of Columbia**	6
**Oklahoma**	6
**Georgia**	6
**Oregon**	7
**Maryland**	7
**Indiana**	7
**Utah**	8
**Alabama**	8
**Virginia**	8
**Wisconsin**	10
**Colorado**	10
**Tennessee**	10
**New Jersey**	10
**Michigan**	11
**Minnesota**	11
**Missouri**	12
**Massachusetts**	14
**Washington**	14
**North Carolina**	14
**Pennsylvania**	15
**Illinois**	18
**Ohio**	18
**New York**	21
**Texas**	37
**Florida**	39
**California**	45

**Fig. 2:**
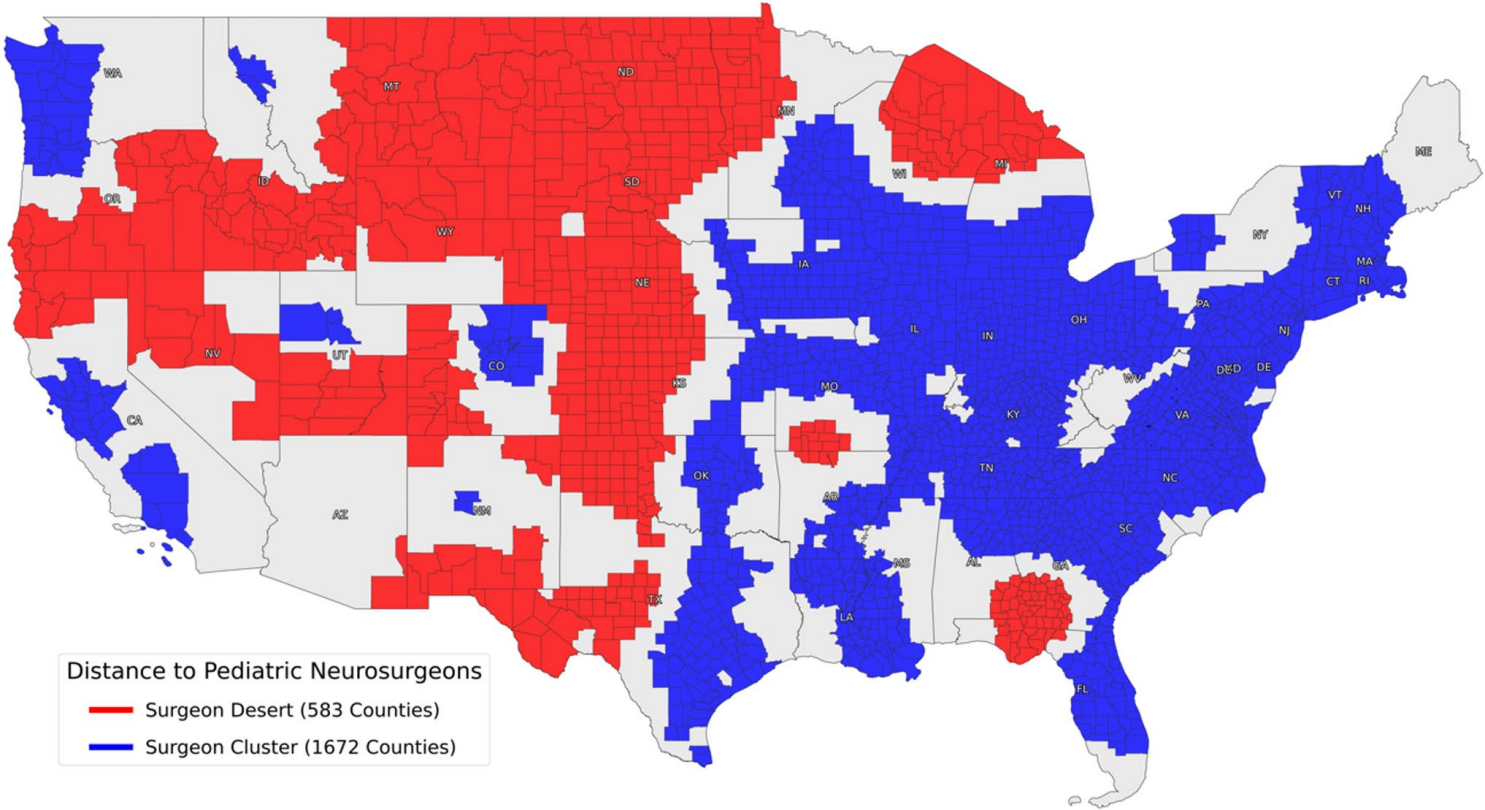
Moran’s *I* cluster analysis of the average distance in miles from any point in a county to the nearest pediatric neurosurgeon displayed on a by-county level. All counties in continental USA were included in the analysis except for a single county in South Dakota (due to missing data)

Figure 2 presents a geographic representation of surgeon deserts and clusters, while Table 2 compares these groups according to socioeconomic, demographic, and pediatric access variables. The average person in a surgeon desert and cluster was found to be 189.2 ± 78.1 miles and 39.7 ± 19.6 miles away, respectively, from the nearest pediatric neurosurgeon (Table 2). Compared to surgeon clusters, surgeon deserts were significantly more likely to be rural (6.96 RUC vs. 3.96 RUC; *p* < 0.001), Republican (62.5% vs. 45.4% Republican voters in 2020 election; *p* < 0.001), comprised of White (87.7% vs. 84.2%; *p* < 0.01), American Indian (5.2% vs. 1.5%; *p* < 0.001), and Hispanic residents (12.1% vs. 7.8%; *p* < 0.001), while less likely to have Black (5.1% vs. 12.2%; *p* < 0.001) and Asian (1.0% vs. 2.2%; *p* < 0.001) residents (Table 2). Residents in surgeon deserts were also found to have significantly lower median household incomes ($52,534.64 vs. $58,027.37; *p* < 0.001), greater percentages of children without health insurance (7.6% vs. 5.3%; *p* < 0.001), and lower percentages of children with disabilities (4.0% vs. 5.0%;* p* < 0.001). The ADI score was also significantly higher in surgeon deserts compared to clusters (68.96 vs. 62.36; *p* < 0.001) (Table 2). Additional comparisons between surgeon deserts and clusters can be found in Supplemental Table 1.

**Table 2:** Bivariate comparison of socioeconomic, demographic, and pediatric access variables between pediatric neurosurgeon deserts and pediatric neurosurgeon clusters

	**Surgeon deserts**		**Surgeon clusters**		***p-value***
**Number of counties**	**583**		**1672**		
**Average variables per county**	**Mean**	**Standard deviation**	**Mean**	**Standard deviation**	
**Geographic access measures**					
**Pediatric neurosurgeons: average county to surgeon distance**	189.29 miles (304.63 km)	78.16 miles (125.79 km)	39.74 miles (63.95 km)	19.66 miles (31.64 km)	** < 0.001**
**Average total pediatric neurosurgeons in a county**	0	0	0.23	1.07	** < 0.001**
**Rurality measures**					
**County population**	23,819.86	56,416.95	149,247.19	404,600.99	** < 0.001**
**County population density**	22.47	56.57	467.05	2445.96	** < 0.001**
**Rural–urban continuum**	6.96	2.15	3.96	2.55	** < 0.001**
**Demographic measures**					
**County median age**	42.44	6.7	41.31	4.78	** < 0.001**
**Percent of county population self identifying as:** **White**	87.73	16.36	84.29	16.23	** < 0.001**
**Black**	5.14	11.61	12.29	15.25	** < 0.001**
**American Indian**	5.25	12.27	1.57	3.2	** < 0.001**
**Asian**	1.09	1.16	2.26	3.51	** < 0.001**
**Hispanic**	12.12	15.87	7.81	10.89	** < 0.001**
**Median household income**	52,534.64	10,326.77	58,027.37	16,424.14	** < 0.001**
**Percent of children without health insurance**	7.66	6.48	5.31	4.32	** < 0.001**
**Percent of children with a disability**	4.04	2.69	5.03	2.13	** < 0.001**
**2020 political leaning (%GOP-%Dem)**	25.15	36.36	− 9.15	46.97	** < 0.001**
**Area deprivation index**	68.96	15.68	62.36	18.4	** < 0.001**

A range of linear regression models with different combinations of covariates were considered to investigate which model showed the best explaining variance of distance to pediatric neurosurgeons. The model with the best goodness of fit showed that higher RUC codes (*p* < 0.001), higher percentages of Republican voters (*p* < 0.001), and higher percentages of American Indian (*p* < 0.001) and Hispanic residents (*p* < 0.001) were independently associated with counties where the average person requires significantly greater travel distances to surgeons. On the other hand, higher percentages of Asian residents and children with disabilities were independently associated with counties where the average person requires significantly less travel distances to surgeons (Table 3).

**Table 3:** Geospatially weighted multivariable linear regression for effect of sociodemographic county characteristics on travel distances to pediatric neurosurgeons

**Covariates**	**Coefficient**	**Lower CI**	**Upper CI**	***p*-value**
**Percent American Indian**	2.08	1.78	2.38	< 0.001
**Percent Asian**	−0.98	−1.98	0.02	0.05
**Percent Hispanic**	0.54	0.40	0.69	< 0.001
**Percent of children with a disability**	−2.37	−3.19	−1.56	< 0.001
**Rural–urban** **continuum**	10.31	9.50	11.11	< 0.001
**2020 political leaning (%GOP—%Dem)**	0.40	0.33	0.48	< 0.001

## Discussion

The current study showed that the average person living in a surgeon desert needs to travel 189.2 ± 78.1 miles to visit the nearest pediatric neurosurgeon, while the average person living in a surgeon cluster only needs to travel 39.7 ± 19.6 miles. Twenty-three states (46%) were found to have fewer than 5 pediatric neurosurgeons, including 4 states with none: North Dakota, Wyoming, Nevada, and Idaho. Our findings are consistent with previous studies. In 2009, Mayer et al. reported that the average distance patients in the USA must travel to meet a pediatric neurosurgeon surgeon was 67.4 miles, with only 25% of hospital referral regions having at least one specialist available [30]. A more recent study similarly demonstrated that the average distance to a pediatric neurosurgeon in the USA was 63.3 miles, with 27.1% of children living more than 60 miles away. They also found that children residing greater than 60 miles away had higher rates of no insurance, families with children living below the federal poverty level, and persons living in rural areas [10]. This trend was consistent with our study: multivariate analyses showed that the average person in counties with higher RUC and ADI scores, higher percentages of Republican voters, and higher percentages of those without health insurance required significantly greater travel distances to pediatric neurosurgeons.

Surgeon deserts had a lower percentage of children with disabilities (4.04%) compared to surgeon deserts (5.03%). One might hypothesize that this is secondary to families intentionally relocating to areas with more accessible specialized care. Census data has shown that roughly 20% of the American population lives in rural areas, but only 9% of the country’s doctors practice there [31]. Unfortunately, over 100 rural hospitals have closed since 2010 because of cutbacks in Medicare and other funding [32]. Similar studies outside the USA looking at neurosurgical access also highlight shortages in rural areas [33–35]. This is important as early diagnosis of pediatric conditions can shape prognosis. For example, adolescent idiopathic scoliosis (AIS) is a pediatric condition that can often be managed non-operatively with bracing if caught early [36, 37]. However, when access to specialists is limited and care is thus delayed, scoliosis curves may be too advanced by the time it is addressed, leaving only operative treatment options.

Numerous factors drive the geographic differences in surgeon supply, but it is still unclear which factors are most influential. Where physicians attended both medical school and residency has been shown to impact final destination of employment. Rosenblatt et al. found that 12 medical schools accounted for greater than one-fourth of the physicians entering rural practice and stated that schools located in rural states were associated with significantly greater rates of rural graduates [38]. Another study queried 2612 randomly selected attending physicians and found that 40% of them moved less than 10 miles from where they trained in residency, and 50% of them moved less than 75 miles [39]. Especially within more specialized fields such as pediatric neurosurgery, physicians are often choosing to practice in highly urbanized areas due to availability of larger academic centers with newer facilities and resources. They also prefer the greater patient volumes, regardless of where they trained [40]. As such, it may be that these surgeon deserts exist in part because they do not reach a critical mass of pediatric patients to support a pediatric neurosurgeon. However, it would be difficult to quantify this critical mass based on our analysis without significant assumptions. In addition, one study in 2005 discussed how physician salaries in rural areas are not significantly higher than those in urbanized areas, despite common perception [41].

The current study also found that higher percentages of White, American Indian, and Hispanic populations were associated with greater distances to pediatric neurosurgeons. Further multivariate analyses demonstrated this to only be true for American Indian and Hispanic populations. One may hypothesize that some findings from our unadjusted analyses are secondary to rural communities being ethnically and racially less diverse [42]. In 2018, the United States Department of Agriculture reported that Whites accounted for 78.2% of rural populations compared to 57.3% of urban populations [43]. It makes sense, therefore, that White residents make up greater percentages of surgeon deserts than clusters due to surgeon deserts being predominantly rural areas. That being said, a report from the CDC in 2017 highlighted that minorities in urbanized centers still face added healthcare access and outcome inequalities [44]. These inequalities are described extensively in both academic literature and modern social culture, yet they remain largely unchanged across several indicators [45–52]. Health-related resources are far from equally distributed, and they are often spaced in a manner that coincides with certain racial and ethnic patterning [2, 3, 53]. This current study presents similar data, showing that counties with greater ADI scores and higher percentages of uninsured residents live significantly further from specialized surgeons—an added barrier to an already challenging path to healthcare access.

With respect to political influences, surgeon deserts were found to have greater percentages of Republican voters, which would align with previous literature. Warraich et al. found that between 2000 and 2019, the mortality gap between Republican-voting counties compared to Democratic-voting counties increased further, especially for White populations [54]. In 2022, Paul et al. also found that Democratic-leaning states had superior outcomes for multiple childhood health measures [55]. However, when it comes to explaining why these trends exist, the story becomes less clear. The Affordable Care Act, passed in 2010, expanded Medicaid and subsequent healthcare access to the degree that significant improvements were made in both inpatient and outpatient surgical outcomes [56]. Since its passage, only 12 states have not yet initiated a Medicaid expansion: Alabama, Florida, Georgia, Kansas, Mississippi, North Carolina, South Carolina, South Dakota, Tennessee, Texas, Wisconsin, and Wyoming [57]. These 12 states, per the most recent Cook Partisan Voting Index, are all Republican leaning; interestingly, 7 of these 12 states were predominantly surgeon clusters [58]. Thus, it is possible that the Republican leanings of surgeon deserts are more a reflection of their rural nature (Table 2), rather than the consequences of Republican control in the state legislature. Although, as Republican leanings were a strong predictor of surgeon deserts even when controlling for rurality (Table 3), it is likely there are other explanations for these findings that are beyond the scope of the variables captured in this analysis. While the correlation between republican voters and surgeon deserts is clear, definitive conclusions on why this is the case have eluded the authors of this work.

An important limitation of this study is that some conditions managed by pediatric neurosurgeons are also treated by pediatric general surgeons, pediatric orthopedic surgeons, adult surgeons, and other physicians without such specialization. Thus, this study only evaluated distances to highly specialized pediatric neurosurgical care. When evaluating population distances from physicians, only the primary locations of surgeon practices were used for analyses, potentially ignoring secondary and tertiary locations where these specialists practiced. That being said, the authors do not believe inclusion of additional sites would meaningfully change our findings as most physician locations are within the same 30 mile radius. Furthermore, data was used from the 2019 United States Census Bureau, indicating the possibility that data has changed over the last 4 years. We therefore believe our findings can appropriately reflect nation-wide trends in the USA, but they are limited in their ability to depict trends beyond this and should be interpreted cautiously.

## Conclusions

Patients residing in counties with greater RUC codes, higher percentages of Republican voters, and higher percentages of American Indian and Hispanic residents on average need to travel significantly greater distances to access pediatric neurosurgeons.

### Supplementary Information

Below is the link to the electronic supplementary material.

Supplementary file1 (DOCX 15 KB)
