# Supplementary material for: Geographic access to pediatric neurosurgeons in the USA: an analysis of sociodemographic factors
Source: Childs Nerv Syst. 2023 Oct 4;40(3):905–12. doi: 10.1007/s00381-023-06172-z (PMC10891277; doi:10.1007/s00381-023-06172-z)
Supplement: Supplementary file 1 — Supplementary file1 (DOCX 15 KB) [file 381_2023_6172_MOESM1_ESM.docx]

**Supplemental Table 1.** Comparison of socioeconomic, demographic, and pediatric access variables between surgeon deserts and surgeon clusters.

|  | **Surgeon Deserts** |  | **Surgeon Clusters** |  | **P-Value** |
| --- | --- | --- | --- | --- | --- |
| **Number of Counties** | **583** | | **1672** | |  |
| **Average Variables per County** | **Mean** | **Standard Deviation** | **Mean** | **Standard Deviation** |  |
| **Population: Under 18** | 5558.84 | 13928.36 | 33299.57 | 91312.2 | **<0.001** |
| **Percent of Housing: Mobile Homes** | 13.04 | 8.87 | 11.48 | 9.34 | **<0.001** |
| **Percent of Housing: Rented** | 27.82 | 8.18 | 28.1 | 8.56 | 0.462 |
| **Percent of Households: Without a Vehicle** | 4.97 | 2.91 | 6.33 | 4.18 | **<0.001** |
| **Percent of Households: Without a Telephone** | 1.98 | 1.93 | 1.86 | 1.11 | **0.001** |
| **Percent of Households: With Children** | 27.89 | 6.69 | 29.52 | 5.02 | **<0.001** |
| **Percent of Households: Not English Speaking** | 10.43 | 12.4 | 8.61 | 9.69 | 0.036 |
| **Percent of Households: Spanish Speaking** | 8.13 | 12.18 | 5.3 | 7.57 | 0.403 |
| **Percent of Households: With a Computer** | 87.51 | 5.39 | 87.94 | 5.5 | 0.039 |
| **Percent of Households: With Internet** | 77.87 | 7.63 | 79.7 | 8.02 | **<0.001** |
| **Percent of Families in Poverty** | 9.9 | 5.99 | 10.05 | 4.96 | 0.573 |
| **Median Rent** | 704.78 | 161.87 | 842.97 | 265.48 | **<0.001** |
| **Unemployment Rate** | 4.38 | 3.27 | 5.2 | 2.02 | **<0.001** |
| **Percent without Health Insurance** | 10.91 | 5.99 | 8.5 | 4.19 | **<0.001** |
| **Average Family Size** | 3.02 | 0.38 | 3.05 | 0.23 | **<0.001** |
| **Percent of 25+ year-olds with GED or Higher** | 88.61 | 6.75 | 87.79 | 5.36 | **<0.001** |
| **Percent of 25+ year-olds with College Degree or Higher** | 22.1 | 7.15 | 23.92 | 10.91 | 0.15 |
| **Percent Veterans** | 8.78 | 2.72 | 8.27 | 2.48 | **<0.001** |
